# Predicting Suicide Attempt Trends in Youth: A Machine Learning Analysis Using Google Trends and Historical Data

**DOI:** 10.3390/jcm14186373

**Published:** 2025-09-10

**Authors:** Zofia Kachlik, Michał Walaszek, Wojciech Nazar, Monika Sokołowska, Aleksander Karbiak, Eliza Pilarska, Wiesław Jerzy Cubała

**Affiliations:** 1 Department of Psychiatry, Faculty of Medicine, Medical University of Gdansk, 80-214 Gdańsk, Poland; michal.walaszek@gumed.edu.pl (M.W.); wojciech.nazar@gumed.edu.pl (W.N.); cubala@gumed.edu.pl (W.J.C.); 2 Faculty of Management and Economics, Gdańsk University of Technology, 80-233 Gdańsk, Poland; monikazsokolowska@gmail.com (M.S.); aleksander.karbiak@gmail.com (A.K.); eliza.pilarska99@gmail.com (E.P.)

**Keywords:** machine learning, suicide, google, online, forecasting, internet

## Abstract

**Background:** Suicide remains a leading cause of death among youth, yet effective tools to predict suicide attempts (SA) in individuals under 18 are scarce. This study aims to develop machine learning (ML) models to predict SA in paediatric populations using Google Trends data. **Methods:** Relative Search Volumes (RSVs) from Google Trends were analysed for terms linked to suicide risk factors. Pearson Correlation Coefficients (PCC) identified terms strongly associated with SA rates. Based on these, several ML models were developed and evaluated, including Random Forest Regression, Support Vector Regression (SVR), XGBoost, and Linear Regression. Model performance was assessed using metrics such as PCC, mean absolute error (MAE), mean squared error (MSE), root mean square error (RMSE), and mean absolute percentage error (MAPE). **Results:** Terms related to suicide prevention and symptoms, including *psychiatrist* and *anxiety disorder*, showed the strongest correlations with SA rates (PCC ≥ 0.90). Random Forest Regression emerged as the top-performing ML model (PCC = 0.953, MAPE = 20.12%, RMSE = 17.21), highlighting *burnout*, *anxiety disorder*, *antidepressants*, and *psychiatrist* as key predictors of SA. Other models’ scores were XGBoost (PCC = 0.446, MAPE = 22.57%, RMSE = 18.03), SVR (PCC = 0.833, MAPE = 42.23%, RMSE = 47.32) and Linear Regression (PCC = 0.947, MAPE = 23.64%, RMSE = 17.66). **Conclusions**: Google Trends–based ML models suggest potential utility for short-term prediction of youth SA. These preliminary findings support the utility of search data in identifying real-time suicide risk in paediatric populations.

## 1. Introduction

Suicide is defined as the act of an individual intentionally ending their own life. The broader term *suicidal behaviour* encompasses a range of thoughts and actions related to this act. These include suicidal ideation (SI)—having thoughts about intentionally taking one’s own life; suicide plan—the development of a specific strategy to achieve so; and suicide attempt (SA)—engaging in potentially self-injurious behaviour with at least some intention to die as a result [1].

Suicide stands as a significant global contributor to mortality, with a particularly concerning impact on the younger demographic. It has emerged as the fourth leading cause of death among individuals aged 15–29 and ranks third among females aged 15–19, as noted in recent global health assessments [2]. The documented average prevalence of active SI in individuals up to 21 years of age is 16.3%, while the prevalence of SA is reported at 6.2% [3]. Recent findings from the National Hospital Ambulatory Medical Care Survey revealed that emergency department visits for SA/SI among the US youth doubled from 2007 to 2015 [4]. Altogether, these data underscore the critical vulnerability of the young population and highlight the importance of early detection and intervention strategies as key components of suicide prevention efforts. 

Despite the urgent necessity for early detection, there remains a significant unmet need for real-time monitoring of suicidal behaviours in adolescents, particularly those occurring in the digital space. The ubiquitous engagement with social media increases their vulnerability to cyberincivility, potentially compromising their mental well-being [5], while severely reduced control over gaming habits, as seen in internet gaming disorder, is closely linked to social anxiety, loneliness, and critically, suicidal ideation [6]. A retrospective postmortem study conducted on South Korean adolescents aged 10–19 who died by suicide identified excessive internet use as a recognisable risk factor for suicide [7]. Conversely, in recent years, the development of digital technologies, such as mental health apps, computerised cognitive behavioural therapy, and AI-driven suicide prevention programs on social media, has increased access to evidence-based interventions for adolescents experiencing mental distress [8,9]. Furthermore, the digital footprint left by individuals in mental health crisis, regardless of its original purpose, coping mechanisms, or exposure to pro-suicidal content, can be measured to provide valuable insights for suicide prevention strategies.

The emerging focus on real-time measurement of SI has found a promising tool in Google Trends, which enables the tracking of suicide-related internet activity to better understand immediate risk and behavioural patterns [10,11]. However, these studies often do not consider age demographics, leaving adolescent-specific risk factors underexplored. A few papers have started to address this gap: one study analysed the correlation between monthly Google searches for known pro-suicide forums and suicide rates in the 10–24 age group [12], while another utilised Naver, Korea’s equivalent of Google Trends, to investigate the relationship between suicide/self-harm-related searches and adolescent suicide rates [13].

These studies, while valuable for identifying patterns of suicide-related search activity, remain limited to descriptive analyses and fail to predict suicide attempts or pinpoint at-risk populations. By integrating Google Trends data with machine learning (ML) techniques, it becomes possible to move beyond observation toward generating predictive insights that support early detection and targeted prevention efforts.

The majority of ML applications in mental health prediction have focused on outcomes such as internet addiction, gambling addiction, and self-injurious behaviour, typically relying on survey or questionnaire-based data rather than internet-derived sources [14,15,16,17]. Nevertheless, recent research has demonstrated the feasibility of using internet-based data to generate real-time ML forecasts of opioid overdose deaths and suicide fatalities [10,11]. However, no studies to date have applied ML models to internet-based data specifically addressing suicidality in youth. Given the growing evidence from studies in the general population, this approach appears to hold considerable promise for identifying patterns and predictors of suicide risk in adolescents [18].

To identify internet search patterns indicating suicide risk among children and adolescents, we first evaluate the correlation between SA rates and Google Trends Relative Search Volume (RSV). RSV represents the standardised popularity of a search term over a given time and location, scaled from 0 to 100, allowing comparisons across different search terms and time periods. This enables us to pinpoint search terms that may serve as key predictors in our machine learning model. Following this, our main objective is to test various machine learning models on the Google Trends data, assessing their efficacy in predicting SA and identifying which predictors hold the greatest significance in the model.

## 2. Methods

### 2.1. Suicide Attempt Data

Our primary outcome of interest was the monthly count of SA (fatal and nonfatal). Data regarding suicide attempts among individuals below 18 years old were provided by the Headquarters of the Polish National Police in the date range from 1 January 2013 to 31 December 2023. A total number of 10,779 SAs were included in the analysis. Reports were generated in one-year resolution, stratified by month and localisation based on supervision of each Polish Voivodeship Police Headquarters, which is highly coherent with the administrative division of Poland. 

### 2.2. Google Trends Search Volume

Our primary input variables were RSVs of several search terms through an open online tool—Google Trends, which reports deidentified data about queries of Google search engine users [19]. RSV is normalised data over selected time frames and localisation in the value range from 0 to 100. The normalised 0 value indicates a very low number of queries in the selected time frame and localisation, and 100 indicates the highest interest. An adjustment process made by Google also includes the exclusion of queries made over a short time from the same IP address [20]. For this study, the region of interest was set to “Poland” (the entire country). The date range was set from 1 January 2013 to 31 December 2023, so that it matched the date range of the suicide attempts dataset.

Firstly, 40 terms related to suicide were selected on the basis of previous studies with a similar subject area [10,13,18,21,22]. Afterwards, those terms were divided into five categories related to suicidal ideations: suicide-seeking, suicide-prevention, suicide-triggers, suicide-symptoms, and psychosis (Table 1). Translations of all terms are presented in Supplementary Material (Table S1). 

### 2.3. Statistical Analysis and Machine Learning Approach

ML models using Relative Search Volumes (RSVs) as input variables were used for the prediction of the number of SA (output—primary outcome). RSVs of all selected terms and categories were tested for Pearson correlation with the suicide attempts count to find the category with the highest correlation. The magnitude of correlation was interpreted based on Pearson and Spearman’s rank correlation coefficient as negligible: 0.00–0.09, weak: 0.10–0.39, moderate: 0.40–0.69, strong: 0.70–0.89, or very strong: 0.90–1.00. The threshold of Pearson or Spearman’s rank correlation coefficient equal to 0.5 or higher was chosen to pre-select the most important input variables (predictors) for machine learning models. In addition, we visually inspected the correlations that were rejected during screening. This analysis did not reveal evidence of relevant non-linear relationships or interaction effects beyond those already captured by the correlation analysis.

Four different machine learning algorithms were tested for SA count prediction from RSVs: Linear Regression, Random Forest Regression, Support Vector Regression, and XGBoost Regression. These four ML models were selected as they present a distinct attitude to model optimisation, and they are extensively used statistical approaches for prediction studies. Before model training, the data were standardised using a standard scaler to ensure comparability of input features across models. As a validation method, we applied 5-fold cross-validation. In this procedure, the dataset was randomly partitioned into five equally sized folds; in each iteration, four folds (80% of the data) were used for model training, and the remaining fold (20%) was used for testing. This process was repeated five times so that each fold served once as a test set, and the final performance metrics were obtained by averaging across all iterations. Importantly, no external hold-out dataset was used for additional validation, and all reported results are based solely on the internal cross-validation procedure.

Statistical efficacy of the models is presented in terms of mean absolute error (MAE), mean square error (MSE), root mean square error (RMSE), and mean absolute percentage error (MAPE). As the Random Forest Models had the best performance from all tested approaches, an additional feature importance analysis was performed to identify the most predictive keywords for the monthly SA rate.

All statistical analysis and ML modelling were performed in Python 3.10 (Python Software Foundation, Beaverton, OR, USA, 2021). using the Pandas library, version 2.1.3 (The Pandas Development Team, NumFOCUS, Austin, TX, USA, 2023), and the Scikit-learn library, version 1.2.1 (Scikit-learn Developers, Inria, Paris, France, 2023).. The threshold of two-sided statistical significance was set at *p* < 0.05 (5%).

## 3. Results

**Figure 1:**
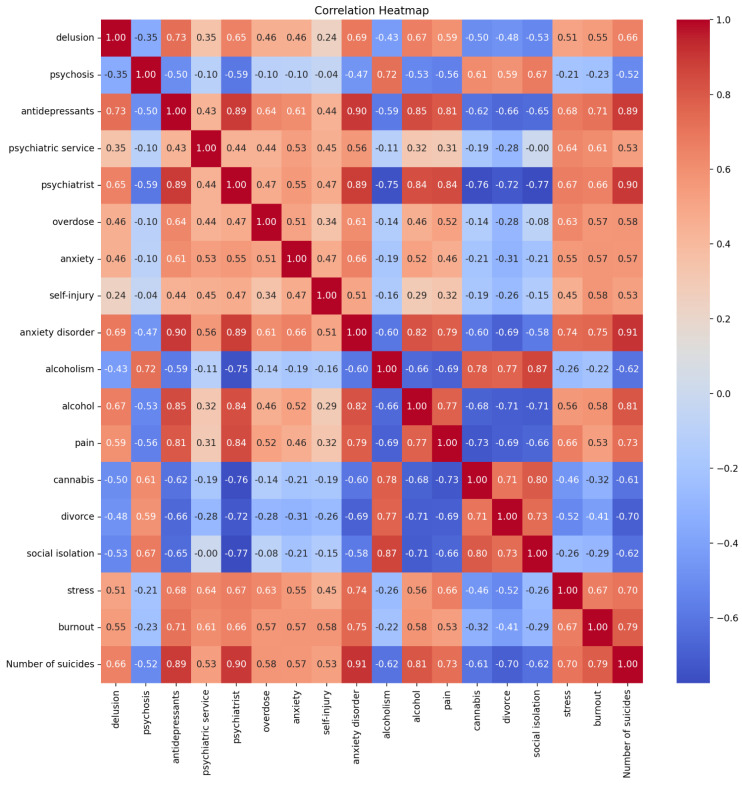
Heatmap presenting correlations between monthly Relative Search Volumes (RSVs) of best predictors and the monthly number of suicide attempts.

Pearson Correlation Coefficients (PCC) for all terms are presented in the form of heatmaps based on categories of terms (Figure S1A–E) and best predictors (Figure 1). Scatter plots for every category group are presented in Supplementary Materials (Figure S2A–E). Categories with a very strong correlation to SA rates include *suicide prevention* (PCC ≥ 0.91). Categories showing a strong association include *suicide symptoms* (PCC ≥ 0.76). In contrast, *psychosis* (PCC ≥ 0.02), *suicide-seeking behaviours* (PCC ≥ 0.04), and *suicide triggers* (PCC ≥ 0.17) demonstrate weak associations with SA rates. In terms of the magnitude of correlation between the number of SA by children and RSVs’ prediction terms, there are two terms with very strong correlation (PCC ≥ 0.90): *psychiatrist* from the suicide-prevention category and *anxiety disorder* from the suicide-symptoms category. Predictors with strong correlation are *antidepressants* from the suicide-prevention category, *alcohol* from the suicide-triggers category, and *pain*, *divorce*, *stress,* and *burnout* from the suicide-triggers category. Negligible magnitude of correlation with predictors’ RSVs is calculated for *bipolar disorder* from the suicide-symptoms category, *mobbing, drunkenness* (both PCC = 0.00), and *sexual abuse* from the suicide-triggers category.

The best predictors for the estimation of children’s SA rate are 17 terms. The terms which met the criteria are burnout, anxiety disorder, antidepressants, psychiatrist, alcohol, stress, cannabis, pain, divorce, psychiatric, service, social isolation, psychosis, delusion, overdose, alcoholism, anxiety, and self-injury. 

**Figure 2:**
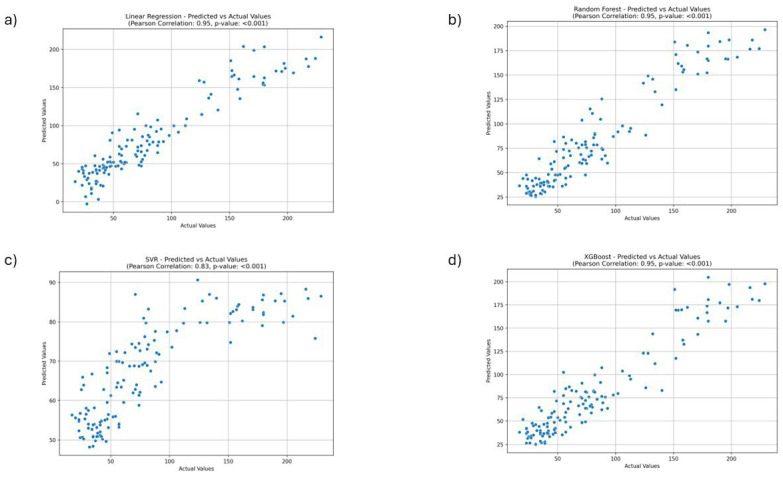
Scatter plots of different machine learning models of suicide attempts, the monthly count of minors aged 18 years old based on Relative Search Volumes. (**a**) Linear Regression, (**b**) Random Forest Regression, (**c**) Support Vector Regression, and (**d**) XGBoost Regression.

**Figure 3:**
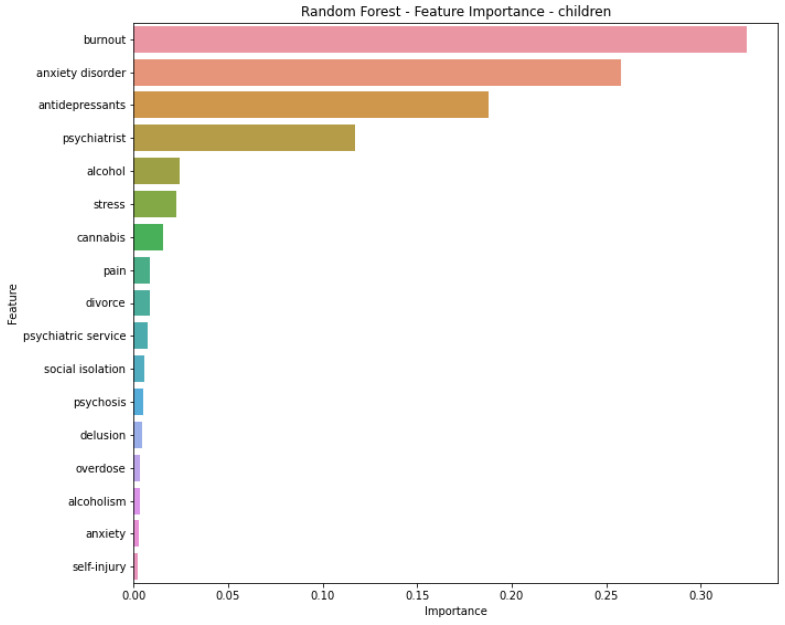
Feature Importance Analysis graph of the performing model—Random Forest Regression.

Table 2 presents a summary of model performance for predicting suicidal attempts by children, and Figure 2 presents scatter plots of the analysed ML models. The ML model that exhibited the best predictive performance was Random Forest Regression, achieving a PCC of 0.953 and an MAPE of 20.12%. In comparison, the SVR model attained a PCC of 0.833 and an MAPE of 42.23%. Both the XGBoost and Linear Regression models also performed well, with PCCs of 0.946 and 0.947, and MAPEs of 22.57% and 23.64%, respectively. Regarding the RMSE, Random Forest Regression had the lowest score of 17.21, followed by Linear Regression with 17.66, XGBoost with 18.03, and SVR with 47.32. The feature importance analysis for the Random Forest Regression model highlighted four significant factors: *burnout*, *anxiety disorder*, *antidepressants*, and *psychiatrist* (Figure 3).

## 4. Discussion

To our knowledge, this study introduces the first ML models aimed at predicting SA in individuals under the age of 18, using Google Trends RSVs as the data source. Our initial analysis established key correlations between SA rates and search terms, showing the *suicide prevention* category had the strongest correlation with suicide attempts (PCC ≥ 0.91), followed by *suicide symptoms* (PCC ≥ 0.76). Notably, terms like *psychiatrist* and *anxiety disorder* demonstrated the highest individual correlation coefficients (PCC ≥ 0.90) within these categories. Moving from correlation to prediction, based on the available data, (1) Random Forest Regression is the most suitable model with the highest PCCs (0.953) and with the MAPE of 20.12%; (2) *antidepressants*, *psychiatrists, anxiety disorder,* and *burnout* are the most significant predictors for SA in our ML model.

This two-step methodology, initially correlating suicide attempt rates with relevant search terms and subsequently developing a predictive correlation-based ML model, sets our study apart from previous research that typically concludes with correlation findings. Therefore, we will leverage the findings from our ML approach and dedicate our discussion entirely to the predictors identified for SA by the established model.

### 4.1. Suicide Risk Factors Among the Youth

Suicide risk factors among the youth show heterogeneity. In children under 10, school-related problems and histories of child maltreatment are prominent risk factors [23]. In adolescents, sexual diversity presents an additional risk factor for SI, compounding the risks that may already be present from earlier developmental stages [24]. Across all youth, a history of mental illness remains a significant predictor of suicide risk. Recent meta-analyses suggest that the risk of SA is particularly elevated in those diagnosed with affective disorders [25]. Depression, the most common affective disorder, often co-occurs with other mental comorbidities. In the Methods to Improve Diagnostic Assessment and Services (MIDAS) project, which studied psychiatric outpatients with major depressive disorder (MDD) as the primary diagnosis, 68.9% were found to have at least one additional psychiatric disorder, including mood, anxiety, substance use, eating, impulse control, and somatic symptoms disorder [26]. Consistent with the aforementioned research, the current machine learning study confirmed the critical importance of psychiatric disorders, as three of our four most significant predictors were associated with psychiatric illnesses. 

### 4.2. Psychiatrist, Antidepressants, Anxiety Disorder

*Psychiatrists* and *antidepressants*, which fall within the suicide-prevention category, may reflect a greater inclination among younger individuals to seek psychiatric support, particularly as the online environment may offer a reduced stigma compared to in-person help. This trend is also reflected in the rising rates of antidepressant prescriptions among children and adolescents in the US, UK, and various European countries [27]. We also propose that the increasing visibility of suicide prevention programs in schools may contribute to young people’s awareness of the value of psychiatric consultation and medication use, subsequently increasing online searches for terms like *psychiatrist* and *antidepressants* [28]. *Anxiety disorder,* classified under the suicide-symptoms category, as mentioned previously, is a common comorbidity of depression [26]. The first population-based longitudinal study on the impact of anxiety disorders on suicide attempts and ideation showed that the coexistence of any anxiety disorder with a mood disorder was associated with a higher likelihood of SA compared to mood disorders alone [29]. Interestingly, our ML model identified the *anxiety disorder* term as a more favourable predictor of SA than the depression-related term. This finding aligns with existing studies across both adolescent and general populations, where no significant association between depression and suicide was observed in the US, Germany, Switzerland, Austria, and South Korea [13,30,31]. Additionally, we hypothesise that individuals with anxiety disorders may exhibit higher Internet engagement and produce a greater volume of RSVs compared to individuals with depressive disorders. This may be attributed to depression symptoms like anhedonia and psychomotor retardation, which may reduce motivation to engage online.

### 4.3. Burnout

One of the surprising findings in our ML study was that *burnout*, classified within the suicide-trigger category, emerged as a significant predictor of SA. Burnout is part of a broader category of stressors, encompassing financial, familial, and occupational stress, that has been previously linked to an increased risk of suicide [32]. Research has associated *burnout* with suicidal risk factors and suicide itself, but these associations primarily focus on work-related burnout within high-demand professions, such as healthcare and emergency services [33,34]. A recent cross-sectional study in a cohort of working individuals in Chile found that both work-related and personal burnout were associated with an increased risk of suicidal ideation and behaviours [35]. We identified one study that analysed search volume data for the term *burnout* among children and adolescents. The stress category, which included burnout, demonstrated a strong correlation with suicide attempts in those under 19 (*p* < 0.01) [36]. Similarly, a previous study examined the relationship between search volume for *dropout* and suicide rates among South Korean adolescents [13]. In fact, the RSV for *dropout*, categorised as a school-related suicide trigger, was positively correlated with suicide rates among males, females, and the total population [13]. *Dropout* and *burnout* share common characteristics, notably their links to stress and overload, feelings of failure and hopelessness, disconnection and isolation, and, most critically, their role as predictors of depression and anxiety. Further evidence of this association comes from an analysis of suicide rates among Japanese children and adolescents, which showed that search terms like “I do not want to go to school” and “study”, both indicative of potential school-related burnout, were statistically significant predictors in a Google Trends model [37]. 

### 4.4. Suicide-Seeking and Psychosis

The *suicide-seeking* category, which specifically includes behaviours associated with actively searching for means of committing suicide, was not a significant factor in our ML modelling. This type of pro-suicidal behaviour often involves access to pro-suicidal websites, but these have largely been successfully blocked or restricted [12], so visiting such sites may no longer serve as a reliable predictor of SA. The *psychosis* category was also not a significant factor in our model. We hypothesise that this may be due to the relatively low prevalence of schizophrenia and other psychotic symptoms among children and adolescents. Epidemiologic studies estimate the global prevalence of early-onset schizophrenia to be approximately 0.5% of the population, which could limit the relevance of this category in predicting suicide attempts among younger populations [38].

### 4.5. Strengths and Limitations

This study has several notable strengths. By integrating Google Trends data with ML methods, we advance beyond the descriptive nature of previous research that relied solely on internet search data to monitor suicide-related behaviour in adolescents. Earlier studies have primarily examined correlations between search activity and suicide rates, which, while informative, provide limited predictive utility. In contrast, our approach leverages real-time, population-level search behaviour to develop predictive models capable of identifying patterns associated with suicide attempts. This study’s findings also offer significant implications for mental health professionals, policymakers, and researchers aiming to prevent suicide among youth. By identifying *burnout*, *anxiety disorder*, *antidepressants*, and *psychiatrists* as prominent predictors, our ML model highlights specific areas where early interventions could be most impactful. The identification of burnout-related terms as a critical predictor, in particular, suggests that school-based programs and mental health interventions should address stress management and resilience-building, targeting not only traditional academic pressures but also the broader psychosocial stresses youth face. The model identifies anxiety-related terms as having higher predictive weight than depression-related ones, which suggests that screening for anxiety symptoms in adolescent populations could be vital for timely intervention. The correlation between psychiatric help-seeking terms, such as *psychiatrist* and *antidepressants*, with SA rates further emphasises the importance of expanding access to psychiatric care and dismantling mental health stigma. This study also illustrates the value of ML approaches in synthesising complex datasets like Google Trends RSVs with real-world suicide attempt data, offering a predictive model that may aid in real-time suicide surveillance. By identifying search patterns that align with periods of increased SA, public health authorities are better positioned to deploy resources or raise awareness during critical times, providing a crucial opportunity for timely intervention.

Despite the strengths described above, this study has several limitations to consider. First, Google Trends RSVs lack demographic specificity (e.g., age, gender), making it difficult to isolate adolescent searches from those of adults. However, prior research validates Google Trends as a proxy for general public interest, suggesting that trends in search volume can still offer valuable insights, even without age specificity [37,39]. Secondly, Google Trends reflects only the queries made through the Google search engine, which may not capture behaviours or search interests of adolescents who are increasingly using other platforms, like TikTok and Instagram. Nonetheless, Google remains one of the most widely used search engines worldwide. Therefore, while it may miss specific platform behaviours, it likely captures a substantial portion of internet search behaviour, particularly on topics as widely discussed as mental health. Thirdly, Google’s normalisation of RSVs from 0 to 100 may obscure absolute shifts in search behaviour, though it still permits analysis of relative changes over time. Thus, we can still observe fluctuations in public interest in suicide-related topics, which is the primary focus of our analysis, rather than exact search counts. Fourthly, although 40 terms were carefully selected based on previous studies [10,18,21,22,36,37], these terms might not encompass all relevant language adolescents might use when discussing or researching suicide online. However, by dividing terms into specific categories (e.g., suicide-prevention, suicide-triggers), we hope to capture a broad range of related searches. Fifthly, ML models may not fully capture the complexity of factors influencing suicide attempts among adolescents, as they rely solely on RSVs and historical data of SA. In fact, previous analysis showed no evidence of association between self-reported anxiety and self-harm and RSVs from Google Trends [40]. While ML models may not capture every complex factor influencing SA, they offer a structured approach to analysing large datasets and identifying patterns that would be difficult to assess manually. Our models, therefore, offer valuable predictions based on available data, even though they do not encompass every variable influencing suicidal behaviour. Sixthly, there may be a lag between when individuals search for suicide-related content online and when SA occurs. Temporal mismatches between searches and real-world events are common in studies using online data, but this does not undermine the overall validity of Google Trends data as an indicator of interest or concern. We designed our models to assess general patterns over time rather than precise day-to-day causality.

## 5. Conclusions

This study provides preliminary evidence of the potential utility of ML models, especially Random Forest Regression, in predicting suicide attempts among individuals under 18 using Google Trends data. With a high PCC = 0.953 and MAPE of 20.12%, our model identifies search terms related to *burnout, anxiety disorders, antidepressants, and psychiatrists* as the most significant predictors for SA in this age group. To our knowledge, this study appears to be among the first to employ an ML approach to identify key suicide-related predictors among adolescents using internet search trends, offering insights that are consistent with risk factors documented in existing descriptive literature. Future research should focus on refining predictor categories, incorporating additional search terms that reflect adolescent language in media content, and exploring alternative platforms popular among adolescents (e.g., TikTok, Instagram, X) to enhance predictive accuracy. These steps may support the development of proactive, data-driven approaches to identify and mitigate suicide risk among young populations. At the same time, it is important to acknowledge limitations such as the lack of demographic specificity, platform exclusivity, normalisation of RSVs, incomplete coverage of adolescent language, model simplifications, and possible temporal mismatches, which together highlight the need for cautious interpretation and continued refinement of this approach.

## Figures and Tables

**Table 1:** Terms selected for assessment as prediction factors are divided into five categories related to suicidal ideations: suicide-seeking, suicide-prevention, suicide-triggers, suicide-symptoms, and psychosis.

Suicide-Seeking	Suicide-Prevention	Suicide-Triggers	Suicide-Symptoms	Psychosis
Suicide	Psychiatric service	Stress	Depression	Schizophrenia
How to kill myself	Psychiatrist	Pain	Self-injury	Psychosis
Poison	Antidepressants	Alcohol	Anxiety disorders	Delusion
Overdose		Alcoholism	Bipolar disorder	Hallucination
		Illicit drugs	I am depressed	
		Cannabis	Phobia	
		Heroine	Anxiety	
		Drunkenness		
		Divorce		
		Violence		
		Unemployment		
		Relationship breakup		
		Cancer		
		Chronic illnesses		
		Burnout		
		Social isolation		
		Separation		
		Sexual abuse		
		Mobbing		

**Table 2:** Presentation of error measures (MAE—mean absolute error, MSE—mean square error, RMSE—root mean square error, MAPE—mean absolute percentage error), PCC—Pearson Correlation Coefficient, actual value (observed outcome from the test fold) and predicted values (model-generated estimate for the same observation based on unseen data) among different machine learning models of suicidal attempts rate.

Model	MAE	RMSE	MSE	MAPE	PCC	*p*-Value	Predicted Values	Actual Value
Linear Regression	14.24	17.66	311.77	23.64	0.947	<0.001	10,708.16	10779
SVR	31.95	47.32	2238.71	42.23	0.833	<0.001	8858.07	
Random Forest	12.69	17.21	296.07	20.12	0.953	<0.001	10,733.16	
XGBoost	14.13	18.03	324.95	22.57	0.946	<0.001	10,548.20	

## Data Availability

The datasets used and/or analysed during the current study are available from the corresponding author on reasonable request.

## Supplementary Materials

The following supporting information can be downloaded at https://www.mdpi.com/article/10.3390/jcm14186373/s1: List of Supplementary Materials includes: Table S1 Translation of potential predictors, Figure S1A Heatmap presenting correlation between relative search volumes of psychosis group terms and suicide attempts count in minor 18 years old, Figure S1B Heatmap presenting correlation between relative search volumes of suicide-prevention group terms and suicide attempts count in minor 18 years old, Figure S1C Heatmap presenting correlation between relative search volumes of suicide-seeking group terms and suicide attempts count minor 18 years old, Figure S1D Heatmap presenting correlation between relative search volumes of suicide-symptoms group terms and suicide attempts count in minor 18 years old, Figure S1E Heatmap presenting correlation between relative search volumes of suicide-triggers group terms and suicide attempts count in minor 18 years old, Figure S2A Scatter plot of Relative Search Volumes (RSVs) of terms from suicide-prevention group and suicide attempts count, Figure S2B Scatter plot of Relative Search Volumes (RSVs) of terms from suicide-seeking group and suicide attempts count, Figure S2C Scatter plot of Relative Search Volumes (RSVs) of terms from suicide-triggers group and suicide attempts count, Figure S2D Scatter plot of Relative Search Volumes (RSVs) of terms from suicide-symptoms group and suicide attempts count, Figure S2E Scatter plot of Relative Search Volumes (RSVs) of terms from psychosis group and suicide attempts count.
