# Supplementary material for: Predicting Suicide Attempt Trends in Youth: A Machine Learning Analysis Using Google Trends and Historical Data
Source: J Clin Med. 2025 Sep 10;14(18):6373. doi: 10.3390/jcm14186373 (PMC12470995; doi:10.3390/jcm14186373)
Supplement: Supplementary file 1 [file jcm-14-06373-s001.zip › Table S1 Translation of potential predictors.pdf]

Table S1 Translation of potential predictors.

**Polish term**	**English term**
Samobójstwo	suicide
jak się zabić	how to kill myself
narkotyki	illicit drugs
marihuana	cannabis
heroina	heroine
depresja	depression
samookaleczenie	self-injury
zaburzenia lękowe	anxiety disorder
choroba afektywna dwubiegunowa	bipolar disorder
schizofrenia	schizophrenia
stres	stress
ból	pain
alkohol	alcohol
alkoholizm	alcoholism
pijaństwo	drunkenness
bezsenność	insomnia
antydepresanty	antidepressants
pomoc psychologiczna	psychiatric service
psychiatra	psychiatrist
terapia	therapy
rozwód	divorce
przemoc	violence
bezrobocie	unemployment
zerwanie	relationship breakup
rak	cancer
choroby przewlekłe	chronic illnesses
mam depresję	I am depressed
psychoza	psychosis
trucizna	poison
przedawkowanie	overdose
omamy	delusion
halucynacje	hallucination
fobia	phobia
lęk	anxiety
wypalenie	burnout
samotność	social isolation
separacja	separation
molestowanie	sexual abuse
mobbing	mobbing
