# Supplementary figures and images for: Predicting Suicide Attempt Trends in Youth: A Machine Learning Analysis Using Google Trends and Historical Data
Source: J Clin Med. 2025 Sep 10;14(18):6373. doi: 10.3390/jcm14186373 (PMC12470995; doi:10.3390/jcm14186373)

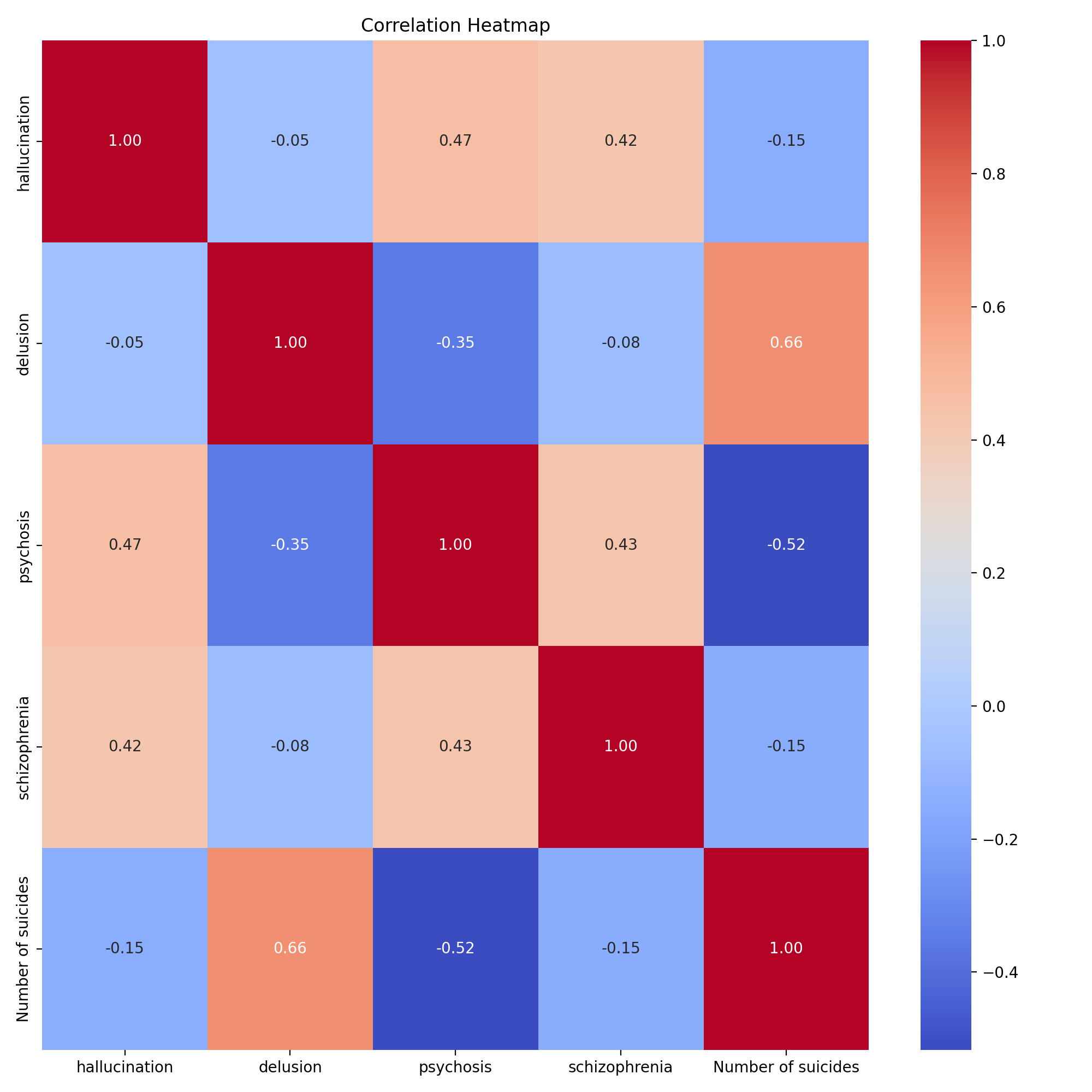

Supplement: Supplementary file 1 [file jcm-14-06373-s001.zip › Figure S1A.png]

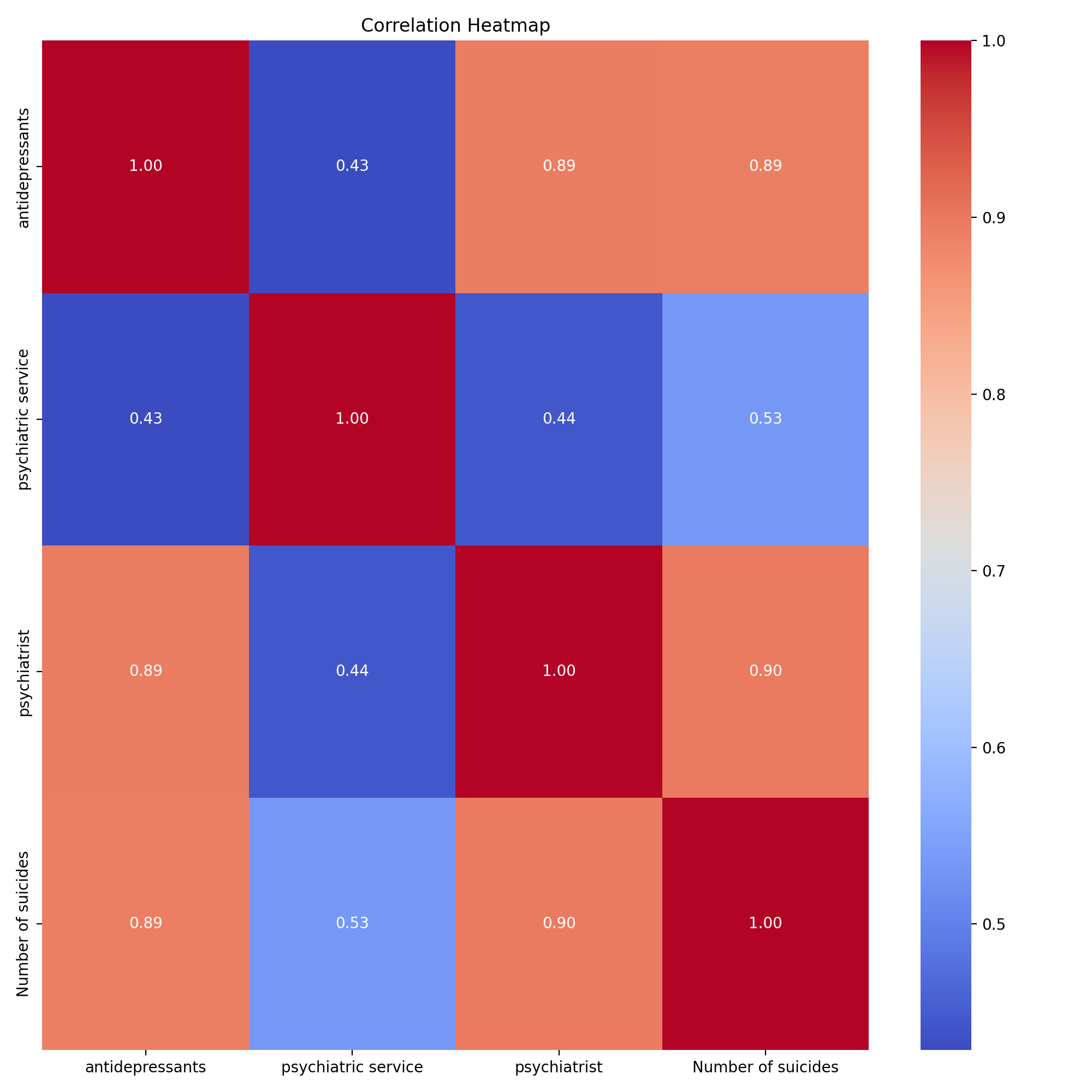

Supplement: Supplementary file 1 [file jcm-14-06373-s001.zip › Figure S1B.png]

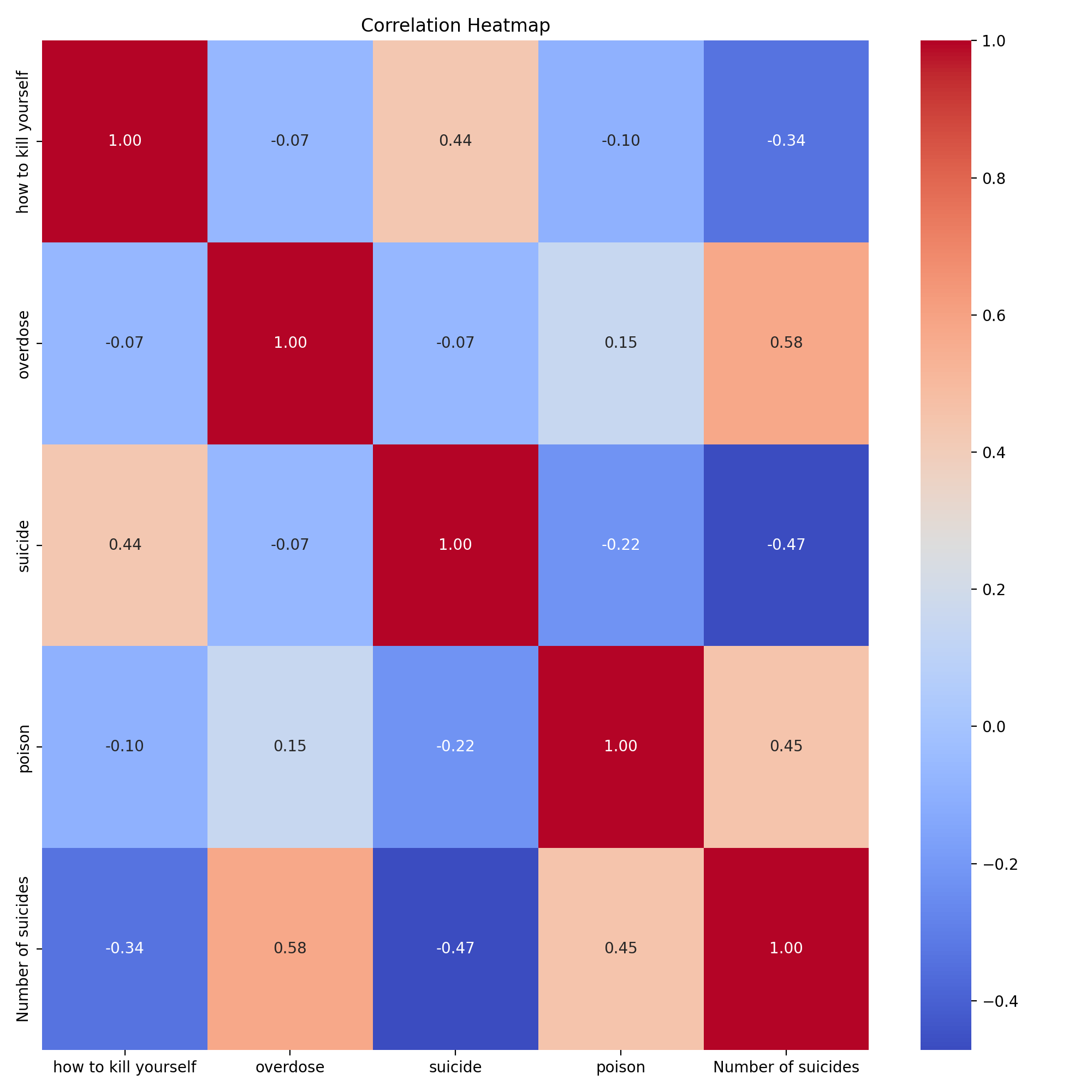

Supplement: Supplementary file 1 [file jcm-14-06373-s001.zip › Figure S1C.png]

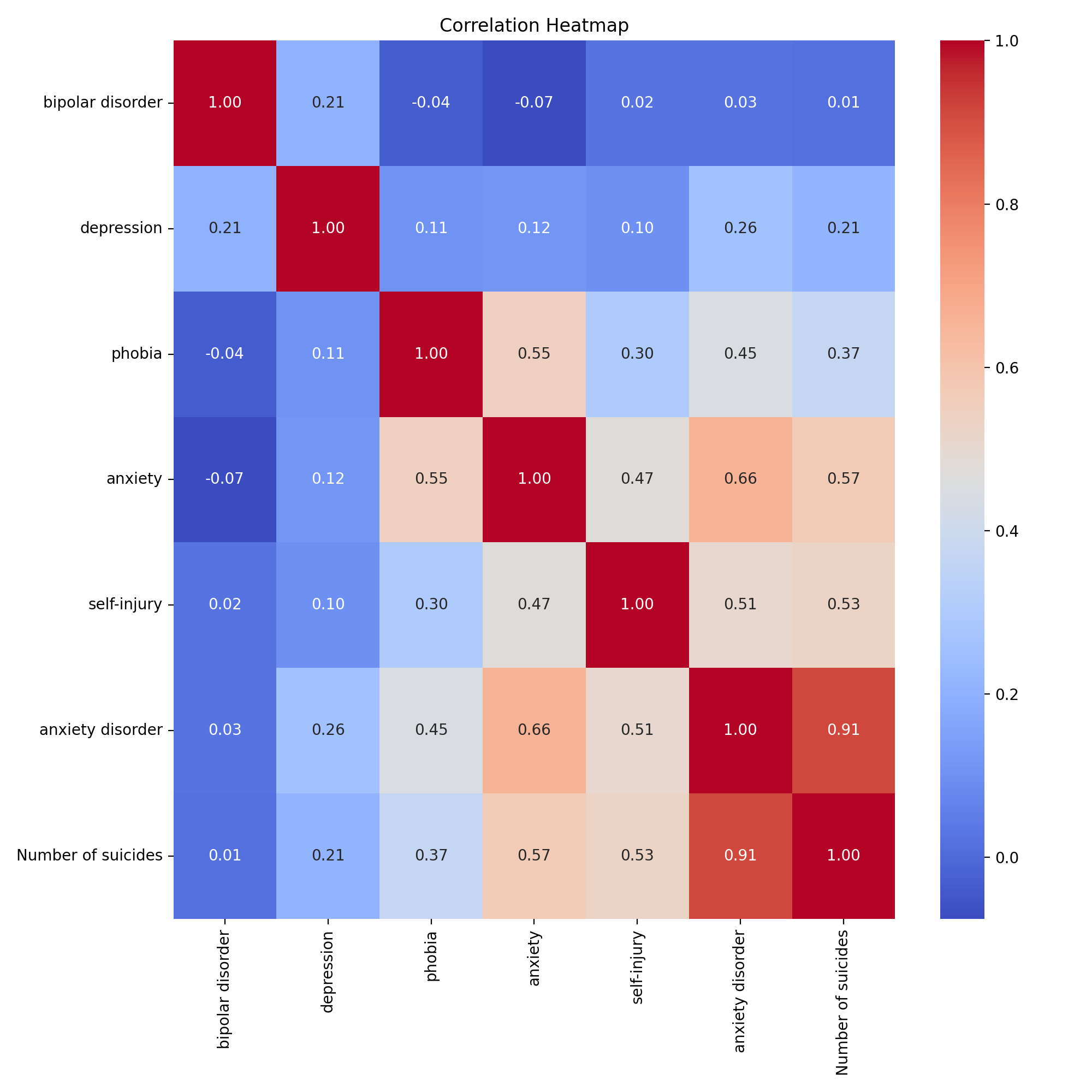

Supplement: Supplementary file 1 [file jcm-14-06373-s001.zip › Figure S1D.png]

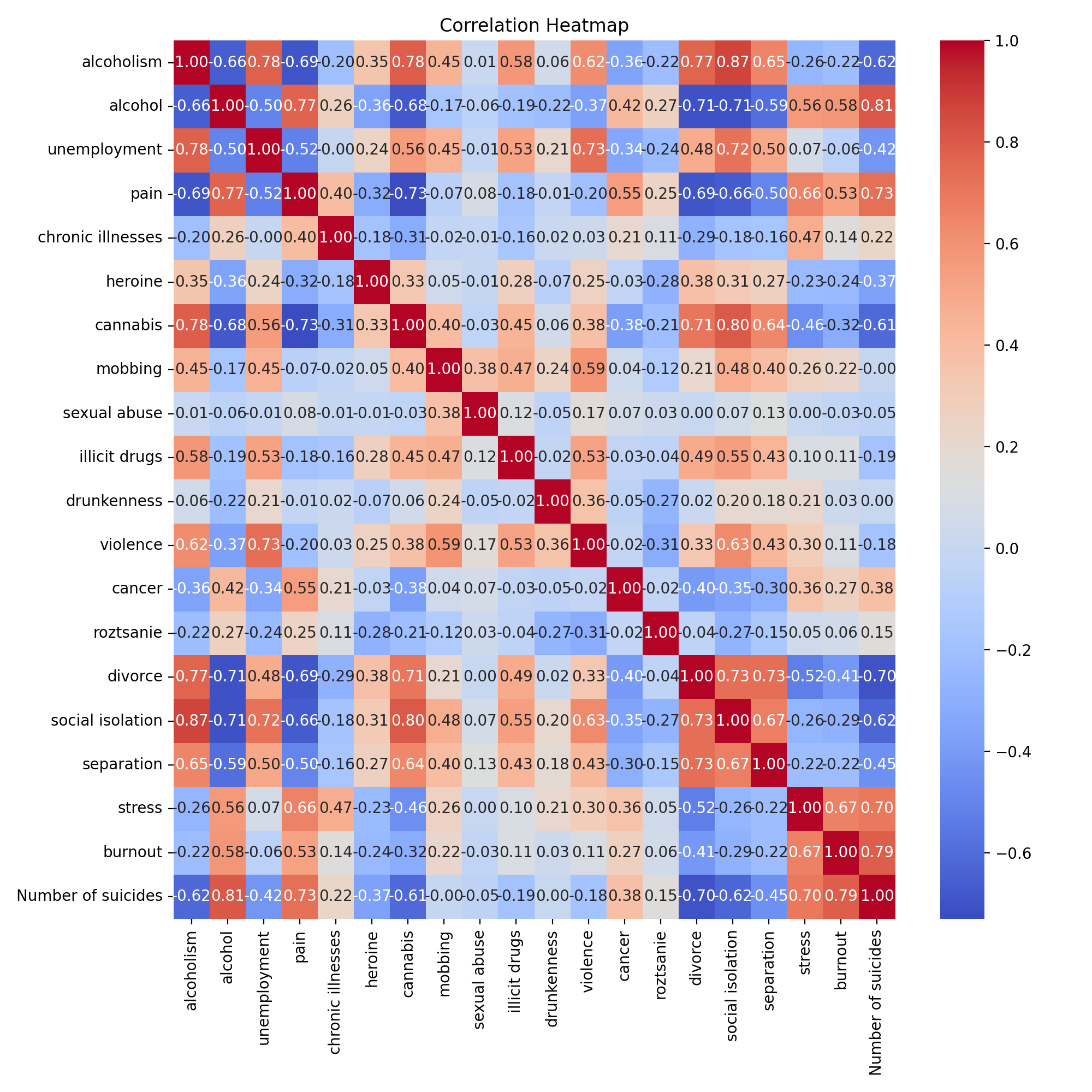

Supplement: Supplementary file 1 [file jcm-14-06373-s001.zip › Figure S1E.png]

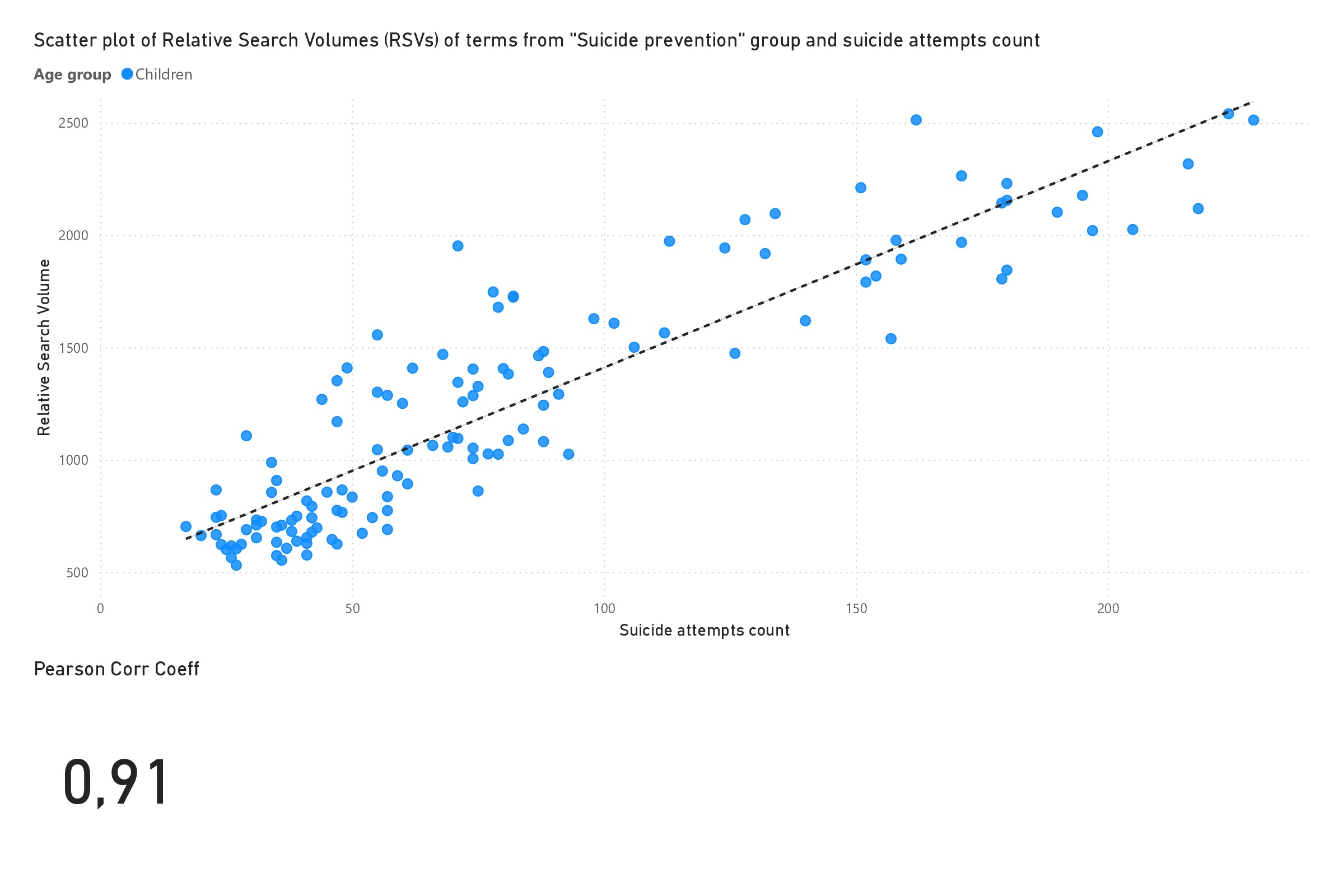

Supplement: Supplementary file 1 [file jcm-14-06373-s001.zip › Figure S2A.jpg]

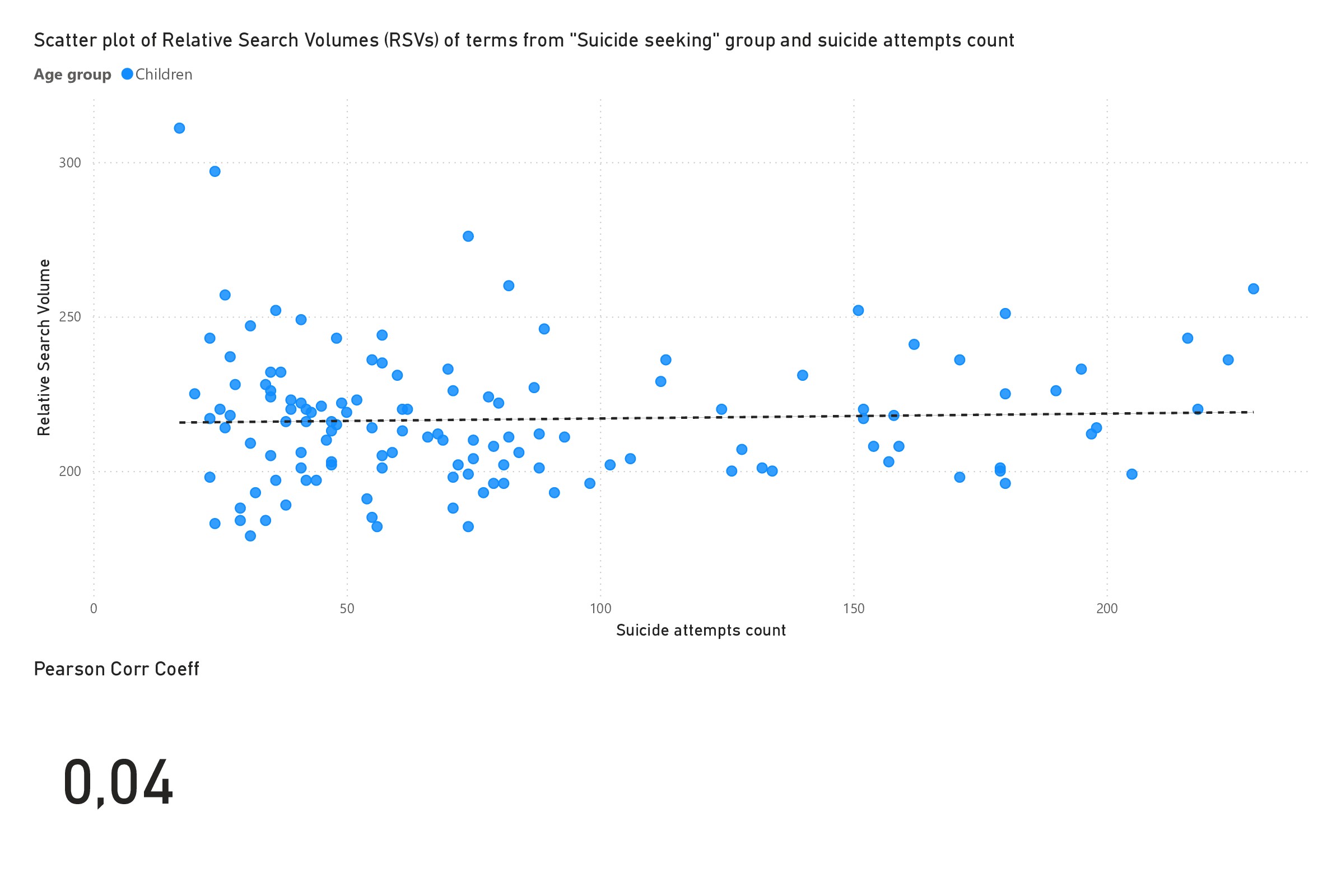

Supplement: Supplementary file 1 [file jcm-14-06373-s001.zip › Figure S2B.jpg]

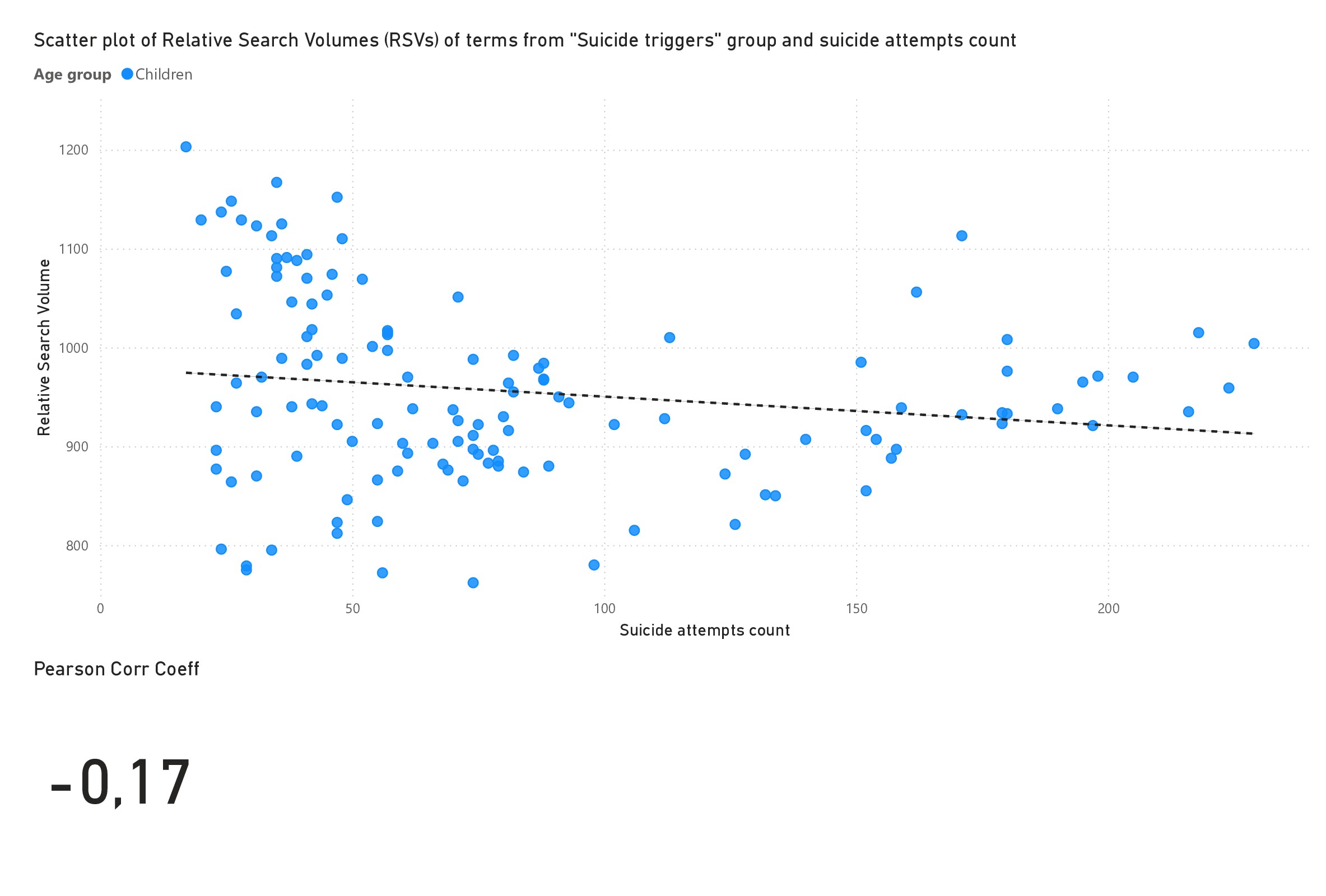

Supplement: Supplementary file 1 [file jcm-14-06373-s001.zip › Figure S2C.jpg]

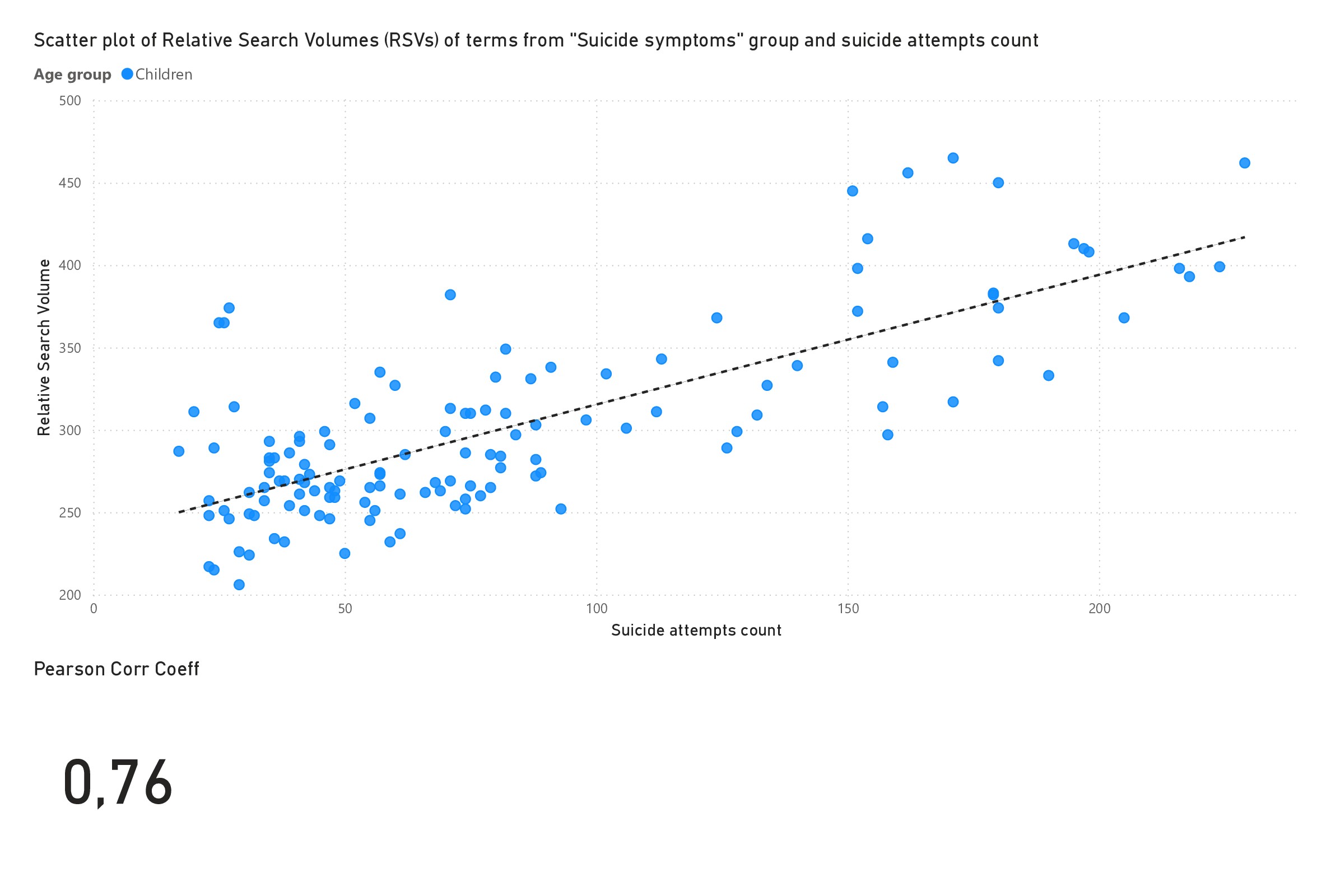

Supplement: Supplementary file 1 [file jcm-14-06373-s001.zip › Figure S2D.jpg]

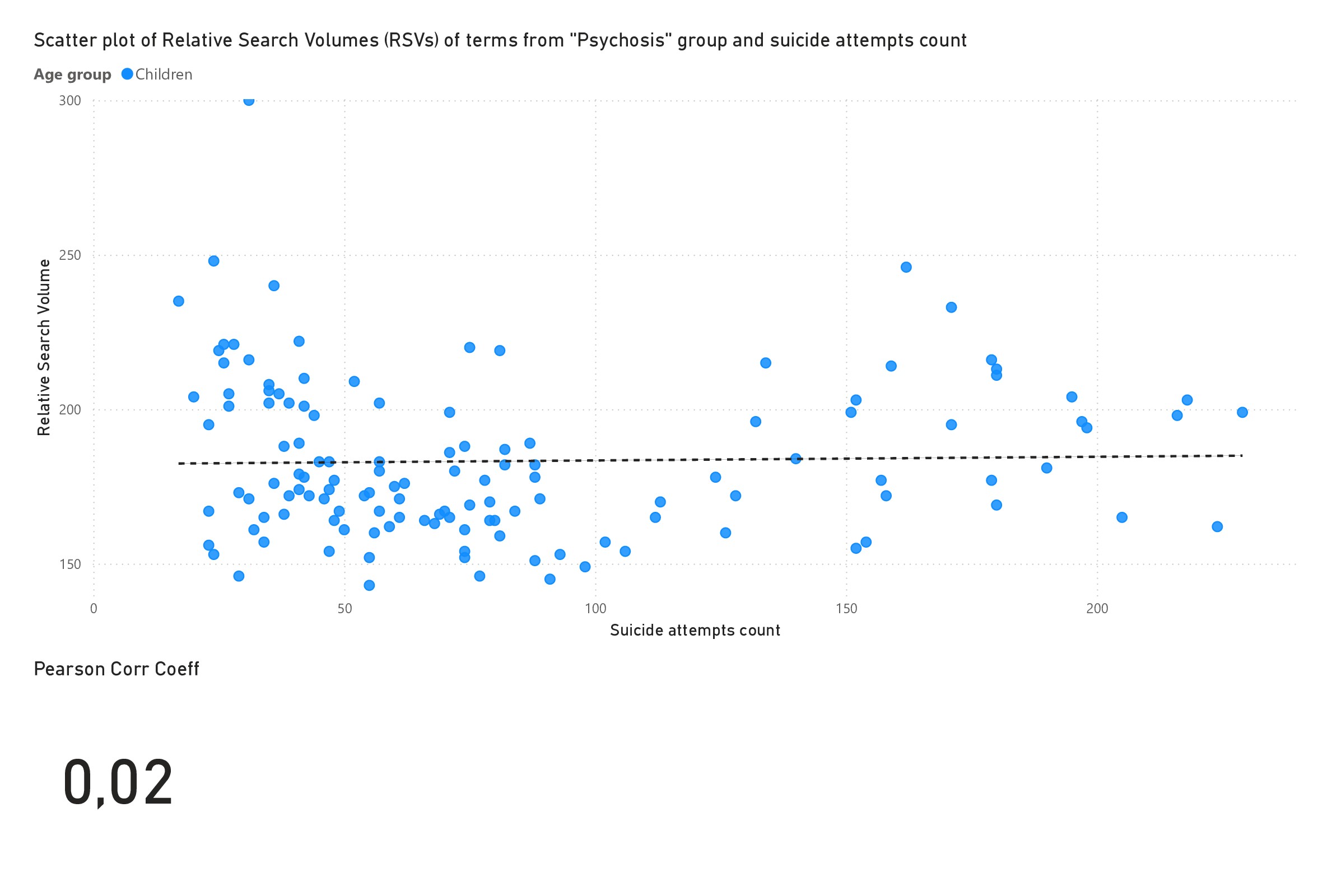

Supplement: Supplementary file 1 [file jcm-14-06373-s001.zip › Figure S2E.jpg]
